# Chemical and Transcriptomic Analysis of Cuticle Lipids under Cold Stress in *Thellungiella salsuginea*

**DOI:** 10.3390/ijms20184519

**Published:** 2019-09-12

**Authors:** Junqing He, Shuai Tang, Di Yang, Yue Chen, Ludi Ling, Yanli Zou, Minqi Zhou, Xiaojing Xu

**Affiliations:** College of Life and Environmental Sciences, Minzu University of China, Beijing 100081, China

**Keywords:** *Thellungiella salsuginea*, cuticular lipids, cold, transcriptome, gene expression

## Abstract

Plant cuticle lipids form outer protective layers to resist environmental stresses; however, the relationship between cuticle properties and cold tolerance is unclear. Here, the extremophyte *Thellungiella salsuginea* was stressed under cold conditions (4 °C) and the cuticle of rosette leaves was examined in terms of epicuticular wax crystal morphology, chemical composition, and cuticle-associated gene expression. The results show that cold induced formation of distinct lamellas within the cuticle ultrastructure. Cold stress caused 14.58% and 12.04% increases in the amount of total waxes and cutin monomer per unit of leaf area, respectively, probably associated with the increase in total fatty acids. The transcriptomic analysis was performed on rosette leaves of *Thellungiella* exposed to cold for 24 h. We analyzed the expression of 72 genes putatively involved in cuticle lipid metabolism, some of which were validated by qRT-PCR (quantitative reverse transcription PCR) after both 24 h and one week of cold exposure. Most cuticle-associated genes exhibited higher expression levels under cold conditions, and some key genes increased more dramatically over the one week than after just 24 h, which could be associated with increased amounts of some cuticle components. These results demonstrate that the cuticle provides some aspects of cold adaptation in *T*. *salsuginea*.

## 1. Introduction

Extreme environmental conditions, including those caused by low temperatures, drought, and salt stress, pose serious threats to agricultural production [1]. Temperature is an important abiotic factor affecting plant physiological processes [2]. Plants can endure or resist low temperature stress by accumulating internal osmotic adjustment substances including proline, betaine, and soluble sugar [3], and increasing the synthesis of metabolism-related proteins, molecular chaperones, and antifreeze proteins [4,5]. Many quantitative trait loci (QTL) and genes were found to be involved in cold tolerance in plants [6,7,8,9,10].

The plant cuticle, covering terrestrial plants, is the first protective barrier against environmental stress [11,12,13]. The cuticle is composed of a cutin polymer matrix, with epicuticular waxes above the cuticle membrane surface and intracuticular waxes embedded within. Wax is a mixture of substances that are soluble in organic solvents, usually including very long chain fatty acids (VLCFAs), alkanes, alcohols, fatty aldehydes, ketones, and esters [14]. Triterpenoids and other minor amounts of sterols and flavonoids also appear in some wax mixtures [15]. Cutin is mainly comprised of C16 and C18 fatty acids and their oxygenated derivatives [16]. The cutin monomers normally include unsubstituted fatty acids, ω- and mid-chain hydroxy and epoxy fatty acids, and α and ω-dicarboxylic acids, and they can have diverse kinds of interlinkages [17]. Variations in cutin composition and ultrastructure have been associated with the physiological functions of cutin [17].

As the outermost layer of the plant, the cuticle plays an important role in plant adaptability to various environmental stresses. Studies have shown that wax is associated with plant adaptability to temperature stress. Temperature variation induced changes in the wax amount and the reorganization of wax crystal structures [18]. Nagpur mandarin (*Citrus reticulata* Blanco) plants subjected to a chilling temperature had wax-coated fruit with less chilling injury [19]. After heterologous expression of two transcription factor genes from *Medicago truncatula*, *WXP1* and *WXP2*, the transgenic *Arabidopsis* exhibited significantly increased cuticular wax deposition on leaves and increased freezing tolerance in the *WXP1* plants with increased alkanes and primary alcohols. The *WXP2* plants, with increased alkanes but decreased primary alcohols, were more sensitive to low temperature [20]. The *sfr3* mutant, with a missense mutation in *ACC1* encoding a multifunctional acetyl-coenzyme A (CoA) carboxylase, exhibited reduced wax deposition on the inflorescence stem and less long-chain wax components on their leaves when grown in the cold compared with wild-type plants and caused freezing sensitivity [21]. During 30 days of storage at 4 °C, the cuticular wax amounts of two blueberry cultivars decreased, and the effect was cultivar-dependent [22]. Therefore, the wax structure and amount are both closely related to cold adaptation in plants, but which wax component plays a key role in cold adaptation is not clear. Compared to wax, much less is known about the cutin layer’s involvement in the cold stress response.

*Thellungiella salsuginea*, which has a heavy coating of surface waxes, exhibits a high tolerance to stress caused by drought, salt, and low temperature [23,24,25,26,27] compared to its mesophytic relative, *Arabidopsis thaliana* [28,29,30]. *T. salsuginea* provides a basis for identifying some valuable genes conferring cuticle traits not apparent in *A. thaliana*, since the genome of *T. salsuginea* has been sequenced [31]. Next-generation sequencing is a recently developed and quick method used to collect transcriptome data. High-throughput RNA sequencing (RNA-Seq) has been used for mining new genes and in molecular regulation mechanism studies, particularly in higher plants under stress. Here, we performed RNA-Seq on rosette leaves from *Thellungiella* plants after exposure to cold (4 °C) and explored cuticle-associated gene expression profiling. To reveal the relationship between cuticle properties and cold tolerance, we also assessed cuticular lipid metabolism in terms of chemical composition. A better understanding of the interactions of the plant epidermal cuticle response to cold environments will provide an improved theoretical basis for the breeding of cold-resistant crop varieties.

## 2. Results

### 2.1. Impact of Cold on the Epicuticular Wax Structure on Leaves of T. salsuginea

**Figure 1:**
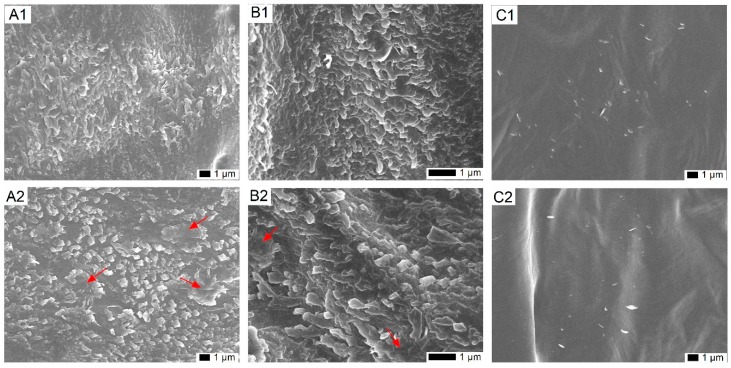
SEM images of the surface wax crystal morphology on rosette leaves of *Thellungiella salsuginea*: (**A1**,**B1**) adaxial and (**C1**) abaxial surface of rosette leaves from untreated plants, and (**A2**,**B2**) adaxial and (**C2**) abaxial surface of rosette leaves from plants subjected to one week of cold treatment. Red arrows indicate aggregated wax crystals after cold treatment.

To examine the change in the wax crystal structure under cold stress, SEM was used to observe the organization of epicuticular wax crystals covered on the rosette leaves of *T. salsuginea*. For leaves from plants in normal growth conditions, wax crystals on adaxial surface have a flattened shape with distinctive lobes in relatively high density (Figure 1A1,B1). Comparatively, after one week of cold treatment, the wax crystal lamellas on the adaxial surface thickened and locally aggregated, but the crystal density was relatively lower than on untreated plants (Figure 1A2,B2). No obvious changes were observed on the abaxial surface after cold treatment, probably due to only few and sparsely distributed wax crystals being produced on the abaxial surface of the rosette leaves of *T. salsuginea* (Figure 1C1,C2).

### 2.2. Impact of Cold Treatment on Cuticular Wax of T. salsuginea

**Figure 2:**
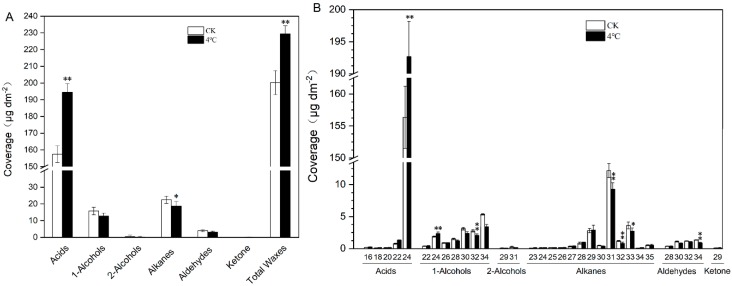
Histograms of (**A**) wax components and (**B**) classes in cold-stressed rosette leaves of *T*. *salsuginea*. The y-axes represent the cuticular wax amount in μg·dm^−2^ of leaf area ± SD (*n* = 5). The x-axes represent cuticular wax components and chain lengths. One-way ANOVA was used to determine statistical differences. * *P* < 0.05, ** *P* < 0.01.

We measured the wax composition of rosette leaves from six-week-old *T*. *salsuginea* plants subjected to 4 °C treatment for one week to assess the impact of low temperature on wax composition. Cold treatment resulted in a significant increase in the amount of total wax and each wax component per unit leaf area. Under 4 °C treatment, total wax was 14.58% higher than on control plants (CK), mainly due to a 23.52% increase in acids. Except for acids and ketones, all other wax components decreased after exposure to cold stress, with the two dominant components, alkanes and primary alcohols, decreasing by 20.49% and 24.45%, respectively (Figure 2A).

In control plants, the acids was the most abundant wax class, accounting for 78.63% of total wax load, followed by alkanes and primary alcohols, accounting for 11.24% and 7.90% of the total wax composition, respectively, followed by aldehyde, and we detected trace amounts of secondary alcohols and ketones. We observed no significant shifts in the proportion of specific wax constituents on the plants subjected to 4 °C treatment (Figure 2A). For 4 °C treated samples, all acid constituents (C16, C18, C20, C22, and C24) increased, with the dominant C24 acid increasing 23.22%. The alkane homologues consisted of the C23–C35 chain constituents, with the dominant C31 alkane decreasing by 23.64%. The primary alcohol homologues consisted of the even-numbered C22–C34 chain constituents, with the dominant C34 primary alcohol decreasing 35.74% (Figure 2B).

### 2.3. Impact of Cold Treatment on Cutin Monomers of T. salsuginea

**Figure 3:**
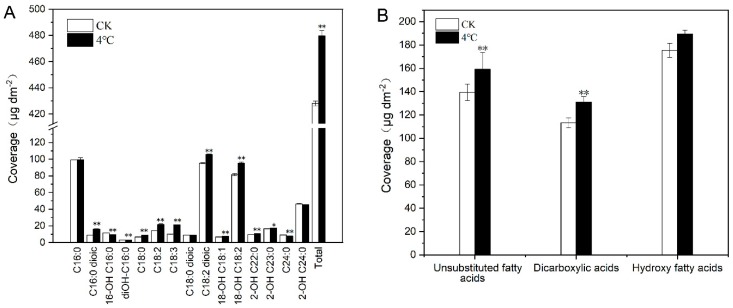
The cutin monomer (**A**) components and (**B**) classes in cold-stressed rosette leaves of *T*. *salsuginea*. Values represent the amount in μg·dm^−2^ of leaf area ± SD (*n* = 5) for each cutin monomer. One-way ANOVA was used to determine statistical differences. * *P* < 0.05, ** *P* < 0.01.

In our study, we identified 15 cutin monomers, with C16:0, C18:2 dioic acids, and 18-OH C18:2 acids being the most abundant components that accounted for more than 50% of the total cutin monomers from leaves of both cold-stressed plants and the CK Compared to CK, the profile of cutin monomers had a little change after one week of 4 °C treatment and the order of four predominate cutin monomers were C18:2 dioic acid, C16:0 acid, 18-OH C18:2 acid, and 2-OH C24:0 acid, with 22.05%, 20.73%, 19.82%, and 9.52% of the total cutin monomers, respectively. The 4 °C stress led to an approximately 12.04% increase in the total cutin monomer amount per unit leaf area. Most cutin monomers increased under 4 °C, particularly 18-OH C18:2, C18:2 dioic acids, and C18:3 acids, which increased by 10.90%, 12.04%, and 110.00%, respectively. Only 16-OH C16:0 acids, diOH-C16:0 acids, and C24:0 and 2-OH C24:0 acids decreased to some degree (15.81%, 10.44%, 12.65%, and 1.73%, respectively; Figure 3A). Cutin monomers can be grouped into three classes: unsubstituted fatty acids, dicarboxylic acids, and hydroxy fatty acids. Unsubstituted fatty acids and dicarboxylic acids all significantly increased under cold stress; however, the increase in hydroxy fatty acids was not obvious (Figure 3B).

### 2.4. Impact of Cold Stress on Total Fatty Acids in T. salsuginea

**Figure 4:**
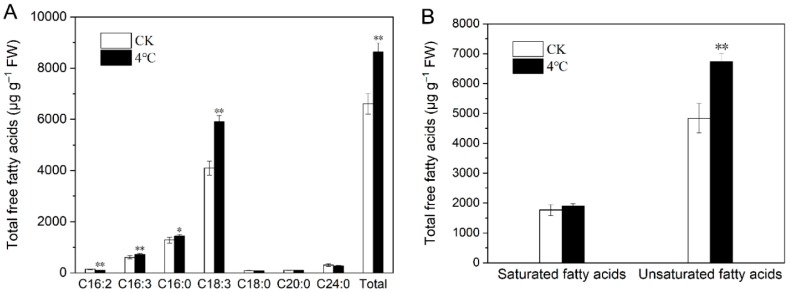
The histogram of the fatty acid (**A**) components and (**B**) classes in rosette leaves of *T*. *salsuginea* under cold stress and the control plants (CK). All fatty acids amounts are expressed as µg·g^−1^ fresh weight (FW) ± SD (*n* = 5). One-way ANOVA was used to determine statistical differences. * *P* < 0.05, ** *P* < 0.01.

Fatty acids have a close relationship with cuticle lipids, which mainly include wax and cutin. C16 and C18 fatty acids are the main common precursors involved in both wax and cutin production. Total fatty acids were assayed from low-temperature-stressed leaves and CK. One week of 4 °C treatment did not change the composition of fatty acids, and seven fatty acid components were identified in total, with C18:3 being the most component in all samples, followed by C16:0. Under cold stress, total fatty acids increased by 38.66% in rosette leaves, with C18:3, C16:3, and C16:0 increasing 55.24%, 24.37%, and 16.24%, respectively. C16:2 was the only component that showed a 23.72% decrease after cold treatment (Figure 4A). Three unsaturated fatty acids components were identified in cold-stressed plants and the CK. The total amount of unsaturated fatty acids and saturated fatty acids increased by 48.94% and 11.38%, respectively, under 4 °C treatment (Figure 4B).

### 2.5. Transcriptome Sequencing and Reads Mapping

To investigate the expression of cuticle-associated genes under low-temperature treatment in *T. salsuginea*, we conducted transcriptome sequencing. Six cDNA libraries were constructed from mRNA isolated from rosette leaves in plants after 24 h of low-temperature treatment and the CK, which were sequenced using BGISEQ-500 RNA-Seq (BGI Biotech Co. Ltd., Beijing, China). The sequencing data were deposited in the Short Read Archive (SRA) database of the National Center for Biotechnology Information (NCBI) under accession number PRJNA533002. Total raw reads accounted for nearly 7 GB of sequencing data in FASTA format. Sequence data were filtered to remove adapters, low-quality sequences, and high contents of unknown bases reads. After quality control, we obtained approximately 23,684,285 and 23,333,838 clean reads from 4 °C treated samples and CK on average, respectively. In total, 96.98% and 90.76% clean reads from cold-treated leaf samples, and 97.11% and 90.02% from CK were mapped to the reference genome and reference genes (Table 1). Sequencing saturation and reads randomness were assessed and the results demonstrated that sequencing data were sufficient for high accuracy functional annotation.

### 2.6. Functional Annotation and Classification of the Differentially Expressed Genes (DEGs)

Compared with the CK, we identified 1195 DEGs, including 844 upregulated and 351 downregulated genes, in rosette leaves in 4 °C stressed plants. These DEGs were assigned into gene ontology (GO) classes, with the molecular function, biological process, and cellular component categories including 741,721, and 628 genes, respectively, which were assigned into 204,598, and 79 GO terms, respectively (Table S1). Many DEGs were found to have different molecular functions and to be involved in various biological processes under low temperatures. Molecular function categories of “binding” and “catalytic activity” were significantly enriched. “Cell part”, “cell”, and “organelle” terms were highly enriched in the cellular component category. “Metabolic process” and “cellular process” terms formed the two largest categories in the biological process category. These GO annotations provided substantial information on the potential functions of these DEGs identified in *T. salsuginea* under low temperature stress. In GO terms, the highest proportion of the 844 upregulated DEGs were classified as “metabolic process”, and the relative proportions of “cell” and “cell part” were second and third highest, respectively. The top three GO terms in the 351 downregulated DEGs were “cell”, “cell part”, and “metabolic process” (Table S2).

**Figure 5:**
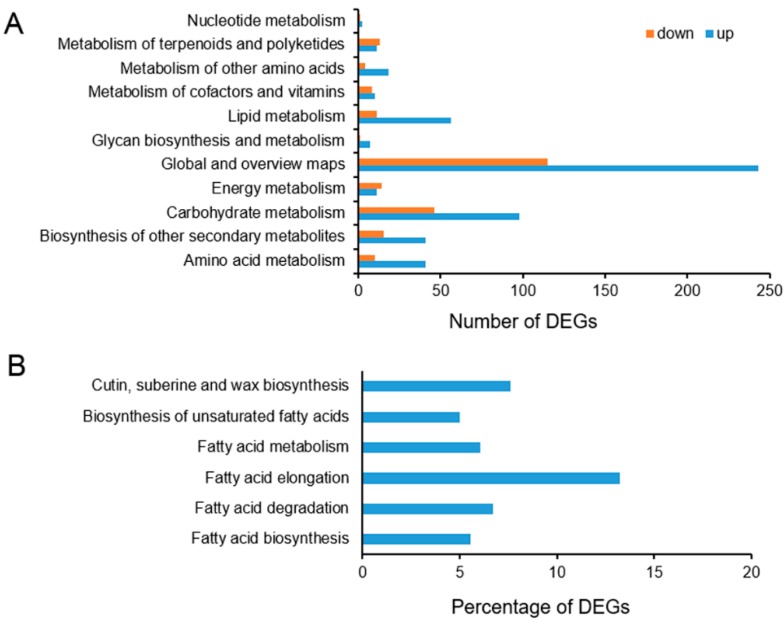
(**A**) The number of differentially expressed genes (DEGs) detected in metabolism pathway and (**B**) the percentage of DEGs with pathway annotation in all genes with pathway annotation putatively associated with fatty acid metabolism of *T*. *salsuginea* under 4 °C stress.

We further analyzed the DEG pathways based on the Kyoto Encyclopedia of Genes and Genomes (KEGG) pathway database. Among the 1195 DEGs, 831 DEGs were found to match the KEGG database and were assigned to 110 pathways. The up- and downregulated DEGs are mainly distributed in the “metabolism” (776), “genetic information processing” (116), and “organism system” (82), accounting for 81.51% of the total pathways. Many of the DEGs were assigned to metabolic pathways (ko01100173) and biosynthesis of secondary metabolites (ko01110129), all belonging to the metabolism category in global and overview maps. Carbohydrate metabolism (144) and lipid metabolism (67) contain the second the third largest numbers of DEGs in the metabolism category, respectively, with carbohydrate metabolism mainly exhibited as starch and sucrose metabolism (ko0050042) and lipid metabolism mainly exhibited as glycerolipid metabolism (ko0056113). The above DEGs were further classified into secondary pathways (Figure 5A; Table S3). Among lipid metabolism, the fatty acid biosynthesis and metabolism pathways involved in cuticle lipid metabolism were searched and the percentage of DEGs with pathway annotation was described. In all six pathways, the DEGs involved represent more than 5% of all genes with pathway annotation. Among these DEGs, the proportion of DEGs involved in the biosynthesis of fatty acid elongation is the highest (13.21%) of all genes with this pathway annotation (Figure 5B).

**Figure 6:**
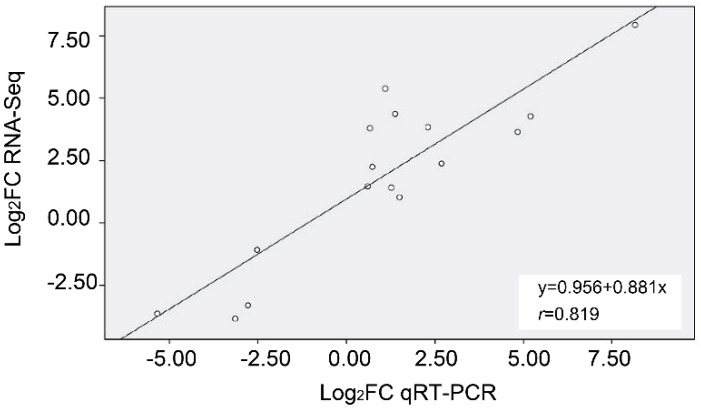
The Pearson correlation coefficient value between the gene expression ratios obtained from RNA-Seq and qRT-PCR analysis of DEGs from low temperature-stress plants.

To validate the transcriptome data, we selected 18 significantly expressed DEGs putatively involved in lipid metabolism and stress response for qRT-PCR analysis (Table S4). The results showed that the trend in gene expression changes determined by RNA-Seq and qRT-PCR under 4 °C treatment versus CK were similar. Correlation analysis was performed on the sequencing data and qRT-PCR, and the correlation coefficients were 0.819. The high correlation coefficient indicates that the transcriptome data we obtained are reliable and efficient (Figure 6).

### 2.7. Impact of Cold on Cuticle-Associated Gene Expression

**Figure 7:**
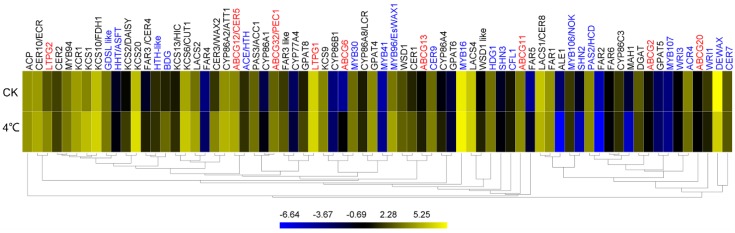
The expression of cuticle-associated genes in the rosette leaves of *T. salsuginea* under cold treatment and the CK, including the genes involved in cuticle synthesis (gene names in black), cuticular lipids exportation (gene names in red), and regulation of cuticle production (gene names in blue). The value on the bar refers to the log_2_FPKM.

Plant cuticle lipids are synthesized through a series of complex biological reactions in which multiple enzymes and proteins are involved. We identified 72 cuticle-associated genes from this transcriptome sequencing and their expression was analyzed based on their FPKM value (Table S5). Among these cuticle-associated genes, 41 genes are involved in cuticle lipid synthesis, nine genes are involved in export of cuticular lipids, and 22 genes are involved in regulation of plant cuticle development. Among 15 genes responsible for VLCFAs biosynthesis, nine genes increased their expression more than 30% (*KCS1*, *KCS2*/*DAISY*, *KCS6,* and *LACS2* increasing more than two-fold) and only *LACS1*/*CER8* and *PAS2*/*HCD* slightly decreased. *FAR3*/*CER4* encoding for acyl-CoA reductase and *WS* encoding for transacylase are the two key genes for production of primary alcohols and wax esters, respectively [32,33]. Eight genes for primary alcohol synthesis were detected and the *FAR1* and *FAR3 like* were found to have relatively high expression levels among these genes in cold-stressed leaves. *FAR1* decreased by 9.78% and *FAR3 like* increased by 90.83% under cold treatment. Three genes for wax-ester synthesis (*DGAT*, *WSD1*, and *WSD1 like*) were assayed, with *DGAT* decreasing by 35.22%, and *WSD1* and *WSD1 like* increasing by 44.55% and 155.84% under 4 °C, respectively. *WAX2*/*CER3*, encoding a putative acyl-CoA reductase, and *CER1,* encoding a putative aldehyde decarboxylase, are involved first in reduction of VLCFAs to aldehydes, leading to formation alkanes, secondary alcohols, and ketones [34,35,36]. Here, two important genes for alkane synthesis, *CER1* and *CER3*/*WAX2*, were all induced under 4 °C, increasing by 164.33% and 372.22%, respectively (Figure 7; Table S5).

The number of genes related to cutin synthesis reported in the literature is much lower than related to wax synthesis. We detected the response of 12 genes putatively associated with cutin synthesis in *T. salsuginea* to low temperature stress. Among these genes, two genes (*CYP86A2*/*ATT1* and *GPAT4*) with high expression levels (FPKM > 20 in control leaves) both increased their level. Similarly, four of five genes (*CYP86A4*, *CYP86A8*/*LCR*, *CYP86C3*, *HTH-like*, and *GPAT8*) with moderate expression levels (FPKM = 1–20 in control leaves) also showed increased expression levels under low temperature, except *CYP86C3* decreased slightly. Among the nine genes related to cuticular lipids export in our study, seven (*ABCG11*, *ABCG12*/*CER5*, *ABCG13*, *ABCG32*/*PEC1*, *ABCG6*, *LTPG1,* and *LTPG2*) exhibited higher transcript abundance under 4 °C treatment. The highly expressed *LTPG1* also increased its expression (Figure 7; Table S5).

We detected the abundance of transcripts of 22 genes putatively involved in cuticle development, including 12 transcription factors and 10 regulatory proteins. *MYB16* and *DEWAX* encoding for MYB transcription factor and AP2 transcription factor, respectively, had the highest expression level in nonstressed leaves (FPKM = 211.22 and 300.13, respectively) and they had different expression patterns under 4 °C, with *MYB16* increasing 6.41% but *DEWAX* decreasing by 6.28%. Among six transcription-factor-encoding genes (*MYB30*, *MYB94*, *MYB96*, *MYB106*, *WRI1*, and *WRI3*) with moderate expression levels, *MYB96* increased significantly (142.74%), whereas *MYB30* and *MYB94* only slightly increased, and *MYB106*, *WRI1*, and *WRI3* decreased slightly. Six of eight regulatory protein encoding genes with moderate expression levels (*ACE*, *BDG*, *CER9*, *CFL1*, *GDSL like*, and *HDG1*) were all induced under cold treatment (Figure 7; Table S5).

The results of cluster analysis showed that genes of *LACS1*, *FAR1*, *PAS2*, *FAR6*, and *DGAT*, involved in wax synthesis, and genes of *CYP86C3*, *ABCG2*, *GPAT5*, and *ABCG20* for cutin synthesis, and some transcription factors genes, such as *MYB106*, *SHN2*, *MYB107*, and *DEWAX*, were clustered together because of their decreased expression under cold stress. Most cuticle associated genes were clustered together due to their increment of gene expression. Wax associated genes of *KCS6*, *LACS6*, *LACS2*, and *CER3*/*WAX2*, one cutin-associated gene of *CYP86A2*/*ATT1*, and cuticle transport gene of *ABCG12* were clustered together. *MYB94* was grouped with some wax-associated genes (*KCR1*, *KCS1* and *KCS10*), and *MYB96* was group with cutin-associated genes (*CYP86A8* and *GPAT4*) (Figure 7).

## 3. Discussion

Cold stress changes the density and the shape and size of wax crystals in stems, and wax load significantly increases after cold stress in glycophyte *Arabidopsis* [37]. The wax load of *Arabidopsis* increases significantly under cold treatment mainly due to the alkanes and 1-alcohols, thus forming a cold-resistant barrier structure and reducing the risk of freezing of cell [37]. *TaFAR4*, encoding a fatty acyl-Co A reductase for primary alcohol biosynthesis, was induced in a cold environment (4 °C) in fruit cuticles of tomato [38]. Here, we found that the density and shape of wax crystals on rosette leaves in *Thellungiella* changed in response to cold stress. *Thellungiella* has a much heavier cuticle than *Arabidopsis*, and *Arabidopsis* do not typically exhibit wax crystals on rosette leaves as does *Thellungiella* rosette leaves. In this study, cold stress significantly increased the leaf cuticle lipid load of *T. salsuginea*, a well-known extremophyte with high tolerance to low temperatures [23]. Comparatively, low temperature led to an increase in the total cuticular wax load in *Thellungiella*, mainly due to fatty acids, the predominant wax class (78.63% of the total wax on normally grown plants), increasing by 23.52%. Acids were previously reported as being critical in forming highly impermeable cuticle barriers [29]. Here, our results show that acids (especially C24 acids) are the most abundant and highly responsive to cold stress in *T. salsuginea*.

Previous studies showed that plants can change their cuticular wax and cutin amounts and compositions to withstand stressful environment, such as water-deficit stress [29,39,40,41,42,43,44], salt stress [43], and abscisic acid application [43]. However, until now, limited research has been conducted on the effect of low temperature stress on plant cuticle lipids, especially on cutin polymers. Our results show that, similar as for the waxes, low temperatures lead to increased total cutin monomer loads, mainly due to the change in the most abundant components, such as 18-OH C18:2 and C18:2 dioic acids, which increased by 16.63% and 16.63%, respectively. Whether these cutin components contribute to the low temperature stress response of the cuticle remains uncertain. In the Shandong ecotype of *T. salsuginea*, these two monomer components increased the most in total cutin monomers on water deficit plants [29]. In *Arabidopsis*, C18:2 dioic, the predominant cutin monomer component, increased by 67% after water deficit [43]. We conclude that 18-OH C18:2 and C18:2 dioic acids may act as key components in forming a cold-resistant cuticle barrier in response to temperature stresses in *T. salsuginea*.

Fatty acids are important precursors for the synthesis of plant cuticle lipids. After 4 °C treatment, the total amount of fatty acids in rosette leaves increased by 38.66%, with combined C16 and C18 fatty acids (the main common precursors for wax or cutin production) increasing 41.36% (Figure 4). The increased fatty acids formed a larger fatty acid precursor pool in accord with the increase in total wax and cutin monomers. C18:3, the most abundant fatty acid component, increased from 61.18% to 68.48% of total fatty acids after cold stress, closely related to the increase in the combined C18 cutin monomers, including 18-OH C18:2 and C18:2 dioic acids, the most two predominant unsaturated cutin monomers. Simultaneously, the increase in C18:3 contributed to the increase in the amount of unsaturated fatty acids, helping to form a membrane structure with better fluidity to resist low temperature stress. We conclude that C18:3 fatty acid is one of key components in the formation of a more cold-resistant cuticle barrier.

From this study, cold stress can not only change the cuticle wax structure and composition, but also the cutin, related to the change in the total fatty acids. To further reveal the effect of cold on the cuticle of *T. salsuginea*, high throughput RNA-Seq was used to analyze the expression level of a list of cuticle-associated genes and their expression change under a low temperature. The association between the expression of cuticle-associated genes and the chemical composition under low temperature stress was investigated. The total amounts of wax and cutin in *T. salsuginea* accumulated to much higher levels under low temperature (4 °C). Among 72 cuticle-associated genes detected in this study, most were induced under the low temperature treatment. Some members from the KCS family, which are involved in the elongation of the precursors of early wax [45], such as *KCS1*, *KCS10*/*FDH1*, *KCS20*, and *KCS6*/*CUT1*, were expressed at relatively high levels and induced under low temperature stress. They possibly play key roles in the production of more wax under low temperature stress. *CER1* and *CER3*/*WAX2*, two key genes for alkanes synthesis [36], were significantly induced under low temperature, in accordance with the alkanes significantly increased under cold stress. MYB transcription factors are generally involved in cuticle development regulation [46]. In this study, *MYB16*, *MYB30*, *MYB94*, and *MYB96* all had relatively high expression levels (Figure 7), and *MYB96* increased its expression level significantly (fold change > 2). MYB96 can regulate LTP3 (a member of the non-specific lipid-transfer protein family presumed involved in export of cuticle lipids from the cell) via direct binding to the promoter, and MYB96 has previously been associated with plant tolerance to freezing and drought stress [47]. In our previous study, we found that several transcription factors had relatively high expression levels in both rosette leaves in before-flowering plants and stem leaves in after-flowering plants of *T. salsuginea*, and even reach much higher levels in stem leaves from after-flowering plants [30]. These transcription factors are considered to play vital roles in maintaining a high content of cuticular wax and cutin in specific tissue and stress responses.

**Figure 8:**
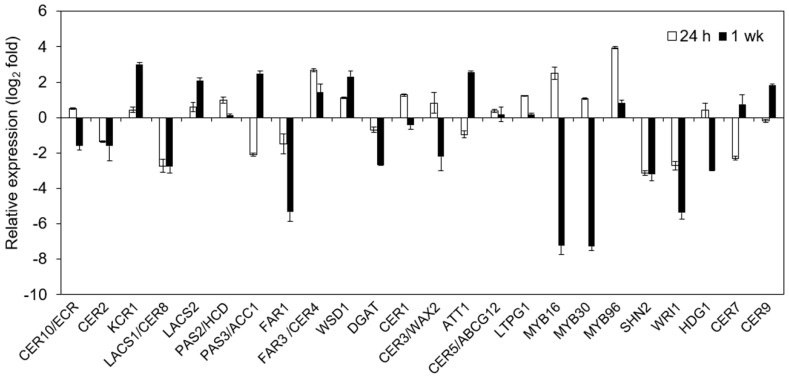
Quantitative RT-PCR analysis of the differential expression of cuticle-associated transcripts in rosette leaves of *T*. *salsuginea* after cold treatment for 24 h and one week.

Cold stress can induce expression of a series of cuticle-associated genes at 24 h of cold stress; however, the accumulation of the chemical components in the cuticle requires much more time than this. Changes in cuticle lipid components were detected after a week or so after abiotic stress treatments [29,43]. Therefore, we selected some cuticle genes to investigate the genes’ expressions under low temperature for one week in addition to 24 h (Figure 8). We assayed the expression of 24 cuticle-associated genes after 24 h and one week of treatment of 4 °C stress. Most genes had similar fold changes in gene expression after 24 h of cold stress using RNA-Seq and qRT-PCR methods (Figure 8, Table S5). The gene expression level after one week of cold stress determined using the qRT-PCR method was compared to that of 24 h of stress. Most cuticle-associated genes showed greater changes in expression after one week of cold treatment than after 24 h cold treatment. Notably, *KCR1* and *LACS2*, two of key genes involved in VLCFA synthesis, increased by 7.04-fold and 3.22-fold after one week of cold treatment compared to 1.41-fold and 0.51-fold increase after 24 h of cold stress, respectively. Another gene involved in VLCFA synthesis, *PAS3*/*ACC1*, increased by 4.64-fold after one week of cold treatment, compared to a 50.98% decrease after 24 h of cold treatment. *WSD1* and *DGAT*, involved in wax-ester synthesis, exhibited a 5.40-fold decrease and 86.60% increase after one week of cold treatment, respectively, compared to the similar change trends but lower change folds after 24 h of stress. *CYP86A2*/*ATT1*, reported to be a key gene for cutin synthesis [48], showed a 5.01-fold increase after one week of cold treatment but a 50.04% decrease after 24 h of cold treatment. Three genes that regulate cuticle development, *CER7*, *CER9*, and *MYB96*, exhibited increased expression levels after one week of cold treatment, but decreased expression levels after 24 h of cold treatment. Two MYB transcription factors (*MYB16* and *MYB30*) and three AP2 transcription factors (*SHN2*, *SHN3*, and *WRI1*) showed significant decreases (more than 16-fold) after one week of cold treatment compared to their up- or downregulated expression after 24 h of treatment. However, *MYB96*, previously reported to be involved in the drought and cold responses [47], still showed increased levels after one week of cold stress. Some genes showed much larger increases after one week of cold treatment compared to decreased expression after 24 h of cold treatment. Some key cuticle-associated genes maintaining higher expression levels up to one week or longer time may be helpful to accumulation of wax and cutin under cold stress in *T. salsuginea*, and these genes are good candidate genes for genetic engineering to improve the cold resistance of crops.

In this study, changes in cuticle composition, structure, and the expression of cuticle-associated genes were observed. We found that cold-induced changes in the cuticle play a role in cold adaptation in *T*. *salsuginea*. The heavier cuticle preserves heat [49] and its structure in response to cold can partly explain the strong cold resistance of *Thellungiella*. However, the relationship between the plant cuticle and cold adaptation indicates the importance of the water retention properties of the cuticle. Being a hydrophobic barrier, the plant cuticle plays a vital role in controlling water loss and improving tolerance to water-deficit environments [50,51]. The heavy cuticle in many broad-leaf evergreens acts as protection from the cold-induced winter drought [52]. Cold stress can damage the cell membrane and lead to cellular dehydration due to the water balance being destroyed with a consequent water loss from the intracellular space to the extracellular space [53,54,55]. However, reduced nonstomatal water loss due to a heavier cuticle barrier can slow the process of cold injury. A freezing sensitivity mutant of *Arabidopsis*, *sfr3,* with a compromised cuticle and reduced wax deposition, has an inability to control the loss of water [21]. Therefore, studying the properties of water retention under cold stress in *Thellungiella* will be necessary in the future. Examining the cold response of the cutin mutant of *Arabidopsis* will also be useful for comprehensively revealing the relationship between the plant cuticle and cold adaptation.

## 4. Materials and Methods

### 4.1. Plant Growth Condition and Low-Temperature Treatment

Seeds of *T*. *salsuginea* (ecotype Shandong) were stratified in the dark for one week at 4 °C. Plants were grown in pots filled with a mixture of vermiculite and soil in a 1:1 ratio and cultured in a growth room at 22 °C under a 16 h photoperiod (90–110 μmol·m^−2^·s^−1^). After two weeks, plants were moved to pots with the same soil mix and cultured under the above culture conditions. Six-week-old (preflowering) *T*. *salsuginea* plants were used for low-temperature treatment. The plants were moved to a cold room at 4 °C under the same light conditions. For transcriptomic analysis, after 24 h of cold treatment, rosette leaves were collected with three replicates with a FW of approximately 500 mg each, with each replicate from five independent plants, and stored at −80 °C after freezing in liquid nitrogen. For chemical analysis, after one week of cold treatment, rosette leaves were collected with 5–6 replicates with 20–25 leaves each (for total fatty acid analysis, 5–6 leaves each), with each replicate sampled from 6–8 independent plants.

### 4.2. Scanning Electron Microscopy

Air-dried rosette leaves were used to view epicuticular wax crystallization patterns on the surfaces by scanning electron microscopy (SEM). Standard sections of leaves between the central vein and leaf margin were sampled for observation using a FEI Quanta 250 SEM (FEI Company, Hillsboro, OR, USA). Leaf samples were prepared and viewed as previously described [56].

### 4.3. Analysis of Surface Cuticular Wax Composition

The lipids were extracted with hexane solvent, and the extracted lipids were defined as surface cuticular waxes and detected according to a previously provided description [35]. We immersed the rosette leaves in hexane for 30 s, followed by a brief 1 s rinse. Wax extracts were evaporated under N_2_ gas and then derivatized in N,O-bis (trimethylsilyl) trifluoroacetamide (BSTFA, Supelco, St. Louis, MO, USA) at 100 °C for 15 min. We analyzed the silylated sample using gas chromatography (GC, SHIMADZU GC-2014, Koyoto, Japan). Helium was used as the carrier gas, and the gas chromatograph used was equipped with a HP-5MS capillary column (30 m, 0.25 mm) (Agilent, Santa Clara, CA, USA) and a flame ionization detector (FID). The initial temperature of the oven was 80 °C, then increased to 260 °C at a rate of 15 °C·min^−1^, maintained at 260 °C for 10 min, then increased from 260 to 320 °C at 5 °C·min^−1^, and held for 24 min under 320 °C. The temperature of both the injector and detector was set to 320 °C. The molecular composition of wax was determined by electron impact GC/mass spectrometry (6890N-5975i, Agilent, Santa Clara, CA, USA). Wax components were quantified based on the FID peak area relative to the internal standard ISTD (hexadecane). Leaf area was calculated using Image J software (Available online: http://rsb.info.nih.gov/ij/).

### 4.4. Analysis of Cutin Monomer Composition

Based on methods previously described [57,58,59], we analyzed the content of cutin monomers in rosette leaves. We soaked the leaves in isopropanol for 2 h then ground them in liquid nitrogen. The ground leaves were incubated in CHCl_3_/CH_3_OH (2:1, *v*/*v*) and CHCl_3_/CH_3_OH (1:2, *v*/*v*) for two days, respectively, and dried in a vacuum desiccator for three days. Depolymerization reactions were conducted at 60 °C for 2 h using 0.9 mL methyl acetate, 1.5 mL sodium methoxide, and 3.6 mL methanol. Methyl heptadecanoate (C17:0 ME) was used as an internal standard. Fatty acid methyl ester was extracted with 10 mL methylene dichloride (CH_2_Cl_2_) and 0.5 mL glacial acetic acid. The organic phase was washed with 0.5 M NaCl three times, and then dried with anhydrous sodium sulfate and evaporated under N_2_. Monomers were derivatized in pyridine and acetic anhydride at 60 °C for 2 h. After cooling to room temperature, the samples were evaporated to dryness under N_2_, and then solubilized in heptane: toluene (1:1, *v*/*v*). The cutin monomers were identified by electron impact GC/mass spectrometry (6890N-5975i, Agilent, Santa Clara, CA, USA) and quantified with GC (SHIMADZU GC-2014, Koyoto, Japan) equipped with FID with an HP-5MS capillary column (30 m, 0.25 mm) (Agilent, Santa Clara, CA, USA). The GC oven had an initial temperature of 800 °C, which was increased to 200 °C at 15 °C·min^−1^, then increased at 1.5 °C·min^−1^ to 230 °C, then increased at 5 °C·min^−1^ to 300 °C. The temperatures of the injector and detector were set to 320 °C. The quantification of the FID peak area relative to the C17:0 ME peak area was determined. Leaf area was determined using Image J software (Available online: http://rsb.info.nih.gov/ij/).

### 4.5. Determination of Total Fatty Acid Profiles

The total fatty acid profiles of *T. salsuginea* leaves were determined as previously described [60], with some modifications. Approximately 50 mg of tissue was placed in the glass tube with a Teflon-lined screw cap (CNW Technologies GmbH, Duesseldorf, Germany) in 2 mL 3% H_2_SO_4_ (*v*/*v*) methanol solution (freshly prepared) and 17:0 ME was added as the internal standard. After 80 °C heating for 1 h, the samples were then cooled to room temperature, 1.0 mL of hexane and 1.5 mL of 0.9% NaCl (*w*/*v*) was added, and fatty acid methyl esters (FAME) were extracted. The sample was shaken violently, centrifuged briefly to facilitate phase separation, and then transferred to an injection vial. The GC procedure was the same as the cutin monomer analysis described above. Molecular identification was completed using electron impact GC/mass spectrometry (6890N-5975i, Agilent, Santa Clara, CA, USA).

### 4.6. RNA Extraction, Library Preparation, and Transcriptome Sequencing

Total RNA was isolated from three different biological replicates for each sample using Trizol reagent (Invitrogen Life Technologies Inc., Carlsbad, CA, USA). RNA quality testing was performed using an Agilent 2100 (Agilent Technologies Inc., Santa Clara, CA, USA), Nano Drop 2000 (Thermo Fisher Scientific Inc., Waltham, MA, USA), and agarose gel electrophoresis. Three replicate RNA samples were used to select mRNA with a poly A tail with oligo (dT) magnetic beads, and then prepared separately for cDNA library construction. The target RNA fragments were reverse transcribed into double-stranded cDNA (ds-cDNA) using N6 random primers. Ds-cDNA ends were repaired with phosphate at the 5’ end, repaired with stickiness ‘A’ at the 3’ end, and an adaptor added with stickiness ‘T’ at 3’ end. Next, the ligation products were amplified with two specific primers. The PCR product was denatured by heating and the single-stranded DNA was circularized by splint oligonucleotides and DNA ligase. Lastly, the cDNA library was sequenced using BGISEQ-500 RNA-Seq (BGI Biotech Co. Ltd., Beijing, China) [61,62]. Sequencing was performed using single end generating 50 bp size reading (SE50). Sequencing data were obtained from the National Biotechnology Information Center (NCBI) short reading archive (SRA; Bethesda, MD, USA) (Accession number: PRJNA533002).

### 4.7. Reads Mapping and Gene Quantification

Low quality reads were removed and clean reads were obtained by filtering the primary sequencing data (raw reads) and then performing quality control (QC) for subsequent analysis. Bowtie2 [63] was used to map clean reads to reference genes and HISAT [64] was used to reference the genome. The alignment results were censused and the random and gene coverages were evaluated. We calculated gene expression levels using the exon model per million mapped reads (FPKM) RSEM method [65].

We screened DEGs from stressed leaves and the CK was performed using the NOISeq method [66]. DEGs were screened according to criteria: fold change ≥ 2 and diverge probability ≥ 0.8. The significant enrichment of the GO function was analyzed using WEGO software to determine the biological function of the DEGs [67]. KEGG was used for analysis of DEG pathway enrichment [68], including metabolic pathways or signal transduction pathways with significant DEG enrichment.

### 4.8. qRT-PCR

Primers were designed from previously reported cDNAs or genes from RNA-Seq data of *T. salsuginea*. We designed gene-specific primers using PRIMER 5.0 software (Table S6). RT-PCR was used to confirm primers quality and PCR amplification efficiency. We used 1 µg total RNA to synthesize first-strand cDNA using the cDNA Synthesis Kit (TransGen Biotech, Beijing, China). Real-time PCR was performed using TransStart^®^ Top Green qPCR SuperMix (TransGen Biotech, Beijing, China) on the Bio-rad system (Hercules, CA, USA). The amplification procedures were as follows: 95 °C for 1 min, followed by 40 cycles at 95 °C for 30 s, 58 °C for 30 s. All samples were assayed in triplicate wells. Data acquisition and analysis were performed for each run using ICYCLERIQ software (version 3.0a Bio-Rad, Hercules, CA, USA). To normalize the differences in the total RNA amount, UBQ5 was used as an internal control [69]. The 2^−△△*C*t^ method was used to calculate the relative expression change [70].

### 4.9. Statistical Analyses

In statistical analysis, Student’s *t*-test in SPSS Statistics 20.0 was used for chemical and gene expression analysis. Five or six replicates were used for chemical analysis, and three replicates were set for gene expression analysis.

## 5. Conclusions

We examined the effect of cold (4 °C) on the cuticle of rosette leaves of the extremophyte *T. salsuginea*. We scanned the surface cuticular wax crystal and analyzed the chemical composition of cuticular wax, cutin monomers, and the total fatty acids. Transcriptome datasets established from rosette leaves from cold-stressed plants and the CK generated basic molecular information for identifying genes involved in cuticle lipid metabolism. We screened 72 unigenes, including 41 genes involved in wax or cutin synthesis, nine genes involved in export of cuticular lipids, 22 genes in cuticle development regulation processes. The results showed that changes occur in the cuticular wax crystal morphology and relatively higher amounts of surface wax and cutin monomers are produced under cold stress, mainly associated with a larger total fatty acid load and higher expression of most of cuticle-associated genes. This study provides information about the effect of low temperature stress on the leaf cuticle of an extremophyte, and the findings help with understanding the cuticle as an adaptation in the low temperature environment response.

## Figures and Tables

**Table 1:** Output statistics from sequencing reads and read alignments.

Sample Name	CK_1	CK_2	CK_3	4 °C_1	4 °C_2	4 °C_3
Raw Data Size (bp)	1,197,620,550	1,162,182,850	1,199,812,800	1,140,096,000	1,178,295,300	1,188,882,800
Raw Reads Number	23,952,411	23,243,657	23,996,256	22,801,920	23,565,906	23,777,656
Clean Data Size (bp)	1,195,529,000	1,159,996,950	1,197,116,750	1,137,586,350	1,176,087,850	1,186,401,500
Clean Reads Number	23,910,580	23,199,939	23,942,335	22,751,727	23,521,757	23,728,030
Clean Data Rate (%)	99.82	99.81	99.77	99.77	99.81	99.79
Clean Reads Q20 (%)	96.80	96.20	96.70	96.00	95.70	96.50
Total Mapped Reads (%) (reference genome/gene)	97.12/89.86	96.89/89.85	97.32/90.35	96.90/90.73	96.87/90.59	97.17/90.96
Unique Match (%) (reference genome/gene)	79.91/75.68	77.53/75.42	79.88/75.52	78.31/78.78	79.52/78.56	80.76/78.71
Multiposition Match (%) (reference genome/gene)	17.21/14.19	19.36/14.43	17.44/14.84	18.59/11.95	17.35/12.03	16.41/12.24
Total Unmapped Reads (%) (reference genome/gene)	2.88/10.14	3.11/10.15	2.68/9.65	3.10/9.27	3.14/9.41	2.83/9.04

## Supplementary Materials

Supplementary materials can be found at https://www.mdpi.com/1422-0067/20/18/4519/s1.

## Abbreviations

BSTFA	N,O-bis(trimethylsilyl)trifluoroacetamide
DEG	Differential expressed gene
FID	Flame ionization detector
FPKM	Fragment per kilobase of exon model per million mapped reads
GC	Gas chromatography
GO	Gene ontology
KEGG	Kyoto encyclopedia of genes and genomes
qRT-PCR	Quantitative reverse transcription RT-PCR
SEM	Scanning electron microscopy
